# Material and Structural Functionalization of Knitted Fabrics for Sportswear

**DOI:** 10.3390/ma15093306

**Published:** 2022-05-05

**Authors:** Ivana Salopek Čubrić, Vesna Marija Potočić Matković, Željka Pavlović, Alenka Pavko Čuden

**Affiliations:** 1 Department of Textile Design and Management, Faculty of Textile Technology, University of Zagreb, Prilaz baruna Filipovića 28a, HR-10000 Zagreb, Croatia; ivana.salopek@ttf.unizg.hr (I.S.Č.); zeljka.pavlovic@ttf.unizg.hr (Ž.P.); 2 Department of Textiles, Graphic Arts and Design, Faculty of Natural Sciences and Engineering, University of Ljubljana, Snežniška 5, SI-1000 Ljubljana, Slovenia; alenka.cuden@ntf.uni-lj.si

**Keywords:** sportswear, knitted fabric, thermal properties, air permeability, comfort

## Abstract

Comfort is an important quality criterion, especially for sportswear. It influences well-being, performance and efficiency. The necessary dissipation of heat and air flow, at high metabolic rates, must be designed and planned in advance. The influence of structure, density, mass and thickness of fabric were considered as well as yarn material composition, yarn linear density, yarn evenness and yarn hairiness. The influence of the mentioned parameters on thermal properties and air permeability was calculated. From the correlation analysis, it can be concluded that yarn’s linear density, yarn short fibers hairiness, and mass per unit area of knitted fabric has the greatest impact on heat resistance. The yarn linear density, the yarn hairiness of the longer protruding fibers, and the thickness of the knitted fabric have the greatest impact on air permeability. A statistically significant model of multiple linear regression equations was offered to predict the thermal comfort of knitted fabric.

## 1. Introduction

Knitwear has always been associated with relaxed clothing and fashion. Now synonymous with the American concept of simple and relaxed casual clothing, sportswear developed as a strong element of the twentieth-century knitwear trade. The simple sweater became emblematic of certain sports like the cricket, golf or sweater [1]. In the Cambridge dictionary, sportswear is defined as “informal clothing designed for comfort” [2], while in Merriam-Webster it is broadly defined as “clothing designed for casual or informal wear” [3]. Recently, a term “athleisure” has also been used, merging the terms “athlete” and “leisure”, meaning “casual clothing designed to be worn both for exercising and for general use” [4] and “a style of clothing that is comfortable and suitable for doing sports, but also fashionable and attractive enough to wear for other activities” [5]. Therefore, the crossover between sport and leisure has been blurred, as sport and leisure are essentially about fun and well-being [6], which is reflected in the terminology as well as in the design and marketing of multifunctional and multipurpose sportswear or athleisure wear.

Today, the blurring boundaries between sportswear and urban wear, and a large number of luxury and high-end ready-to-wear brands developing sports-inspired ranges, are a growing source of novel design orientations for performance clothing. Sports-inspired garments are now standard everyday wear, especially among younger generations, defining what consumers will wear in the future. The interactions between these two areas of influence, sports and fashion, are an important source of new trends in both industries. As for sports companies, a large part of their business is selling after-sports apparel to their core clientele. They are also trying to attract a broader clientele that is looking for a sporty silhouette but not necessarily engaged in a specific sporting activity [6].

The reason for the merging of sportswear and casual or urban fashion could be the dramatic increase in participation in sports activities and interest in health and fitness in the last three decades of the twentieth century, which has expanded the market for sports-specific apparel. The global sportswear market is expected to grow at a CAGR of 5.8% over the next five years. At the same time, the share of sports apparel in amateur sports is around 85%. Performance sportswear has become increasingly sophisticated in terms of styling and detailing, benefiting from rapid developments in fibre and fabric technology and modern garment construction methods. These influences have been adopted in products ranging from extreme sports to related market sectors such as adventure travel, corporate wear and health. It can be said that sportswear is becoming a very popular style statement and fashion trend [6,7].

Sportswear has, in a sense, come full circle from the influence of sportswear on high fashion and streetwear, to the influence of streetwear on the design and marketing of the collections of sportswear companies. Today, for example, many major ski-wear brands have launched their own urban collections, adding a Nordic high-fashion twist to technical outdoor designs [8]. People like to wear sportswear, which means they want to feel comfortable in it.

Comfort is an important quality criterion. It influences not only the wearer’s well-being but also their performance and efficiency. Comfort is also an important sales aspect [6]. However, it is very complex and difficult to define. According to Fourt and Hollies [9], comfort includes thermal and non-thermal components and is related to wearing situations such as work, non-critical and critical conditions. The physiological responses of the human body to a given combination of clothing and environmental conditions are predictable when the system reaches a steady state. According to Slater [10], comfort is a pleasant state of physiological, psychological, neurophysiological and physical harmony between the human and the environment.

Modern knitwear encompasses a wide range of products, from functional and protective workwear and activewear for sports or other physical activities that border on technical textiles in their properties, to sidewalk and high-fashion knitwear with distinct esthetic value and many intermediate variations. In all of them, thermal comfort is a very important property that must be designed and planned in advance. This can be achieved through the material, mechanical and chemical functionalization of the knitted structure and style.

## 2. Thermal Comfort of Knitted Sportswear

Thermal comfort is the state of mind that expresses satisfaction with the thermal environment, which means that a person feels neither too cold nor too hot [11]. As an interface between the human body and the environment, textiles play an important role in the heat exchange between the body and the environment [12]. The contribution of clothing to thermal comfort is to assist the human body in maintaining comfortable thermophysiological conditions in a wide range of environments. The thermophysiological comfort of clothing is therefore the ability of clothing to assist the human body in maintaining thermal comfort [13]. From a functional point of view, comfort includes a suitable shape and fit that provides freedom of movement, thermal regulation with moisture-wicking and quick-drying properties, and lightweight protection and durability. A suitable mix of all these aspects contributes to an overall “feel good factor” for the wearer [14].

The fabrics for activewear and sportswear are specially designed to achieve the necessary dissipation of heat and moisture at high metabolic rates, both in terms of geometry, packing density and the structure of the constituent fibres in yarns as well as in terms of the structure of the fabric [6].

The thermal insulation of a fabric is determined as its barrier function or resistance to heat transport. Therefore, insulation is physically defined as the thermal resistance of the fabric. At a steady state, there is a temperature difference between the two sides of the fabric and a defined heat flow through the fabric. Thus, the thermal resistance, usually referred to as *R_ct_* (in m^2^ KW^−1^), can be determined using the following equation:

(1) $$R_{ct}=\frac{\left(T_{s}-T_{a}\right)}{Q}\;\cdot A$$

where *T_s_* is the temperature at the skin surface (in K); *T_a_* is the temperature of the environment (in K); *Q* is the heat flux through the fabric (in W); *A* is the area of the fabric (in m^2^).

The thermal properties of clothing, especially high-performance, athletic, or undergarments, are critical to the comfort of the wearer. Many researchers have studied the relationship between thermal resistance and the structural characteristics of fabrics, such as structure/texture, thickness, mass per unit area, thread or loop density in woven and knitted fabrics, loop length in knitted fabrics, etc., and have focused on the association of thermal properties with the properties of specific structures, yarns and fibres.

The influence of different knitted structures, namely single jersey, 1 × 1 rib and interlock, was investigated by Oglakcioglu and Marmarali. The results showed that double jersey structures may be preferred for winter garments to protect against cold due to their high thermal insulation values. 1 × 1 rib fabrics are still more commonly used due to their warmer feel on first contact. On the other hand, single jersey structures should be chosen for active sports or summer clothing due to their better moisture management properties [15]. Amber et al. investigated the thermal properties and moisture transport of socks knitted in single jersey, half terry and terry structures that differed in fibre type and yarn structure. The results indicate that, apart from the effects on regain, the fibre effects are generally small and probably masked by the effects of fabric structure [16]. Uçar and Yilmaz analysed the natural and forced convective heat transfer properties of 1 × 1, 2 × 2 and 3 × 3 rib knitted fabrics from acrylic yarns. It was found that a decrease in the number of ribs led to a decrease in heat loss due to an increase in the amount of air trapped between the front and reverse loops. The results also showed that as the tightness of the fabric increases, the heat loss decreases due to lower air permeability [17]. Erdumlu and Saricam investigated the thermal comfort properties of flat knitted fabrics with different structures, tightness, thickness and porosity made of acrylic fibres specifically designed for winter clothing. Thermoregulatory properties, breathability and thermophysiological properties and their relationship with the fabric’s structural parameters were investigated. The results showed that 2 × 2 rib structures provide the optimal conditions in terms of thermoregulation, breathability and thermophysiological comfort. The thickness and porosity of the fabrics should be adjusted accordingly, as thickness improves thermal insulation and porosity improves breathability [18]. The effect of position and number of tuck loops on the thermal comfort of knitted fabrics was studied by Senthilkumar and Suganthi. It was found that the greater the distance between successive tuck points, the better the air, heat and moisture transfer properties. Bi-layer knitted fabrics with loose structure allowed less thickness and mass per unit area and exhibited better thermal comfort properties [19]. Yang et al. studied bi-layer knitted structures made from filament yarns on a circular knitting machine. It was found that bi-layer knitted fabrics with meshes on one side had better air permeability, moisture management, drying performance and thermo-physiological properties than bi-layer knitted fabrics with trim and symmetrical structure without meshes. Fabrics composed of yarns with finer fibers exhibited better thermal comfort properties [20].

In addition to the structure itself, researchers have also focused on investigating other properties of knitwear that may be related to thermal properties. Ozdil et al. evaluated the influence of yarn count, twist coefficient, combing process and tightness on the thermal properties of 1 × 1 cotton rib fabrics. It was found that with the increase in yarn twist, the thermal absorptivity and water vapour permeability of the fabrics also increased. Higher thermal absorptivity gives the fabric a cooler feel. Thermal resistance decreases as the yarn twist coefficient increases. The effect of yarn twist on fabric conductivity is insignificant. Thermal resistance is lower in fabrics made of combed yarns, while thermal conductivity, thermal absorbency and water vapour permeability are higher compared to fabrics made of carded yarns [21]. Prakash et al. studied the effect of loop length on thermal comfort properties of bamboo knitted fabrics and observed an increase in air and water vapour permeability with a decrease in yarn linear density and an increase in loop length [22]. Mishra et al. investigated the thermo-physiological comfort properties of single knit fabrics and their derivatives made from 100% cotton yarns at three different yarn linear densities and after different relaxation stages. It was found that the physical properties of the fabric were affected by the change in linear density of the yarn and by the dry or wet relaxation stages. The percentage or number of tuck stitches, the location of tuck stitches, and the ratio of tuck stitches to knit stitches have a strong influence on the physical and thermo physiological properties of single knitted fabrics, even when the other knitting parameters remained the same [23].

Other researchers investigated the influence of different fibre and yarn types on thermal comfort. Oglakcioglu et al. investigated the properties of single jersey knitted structures made with channeled and hollow polyester fibres. The results showed that channeled PES fabrics are suitable for hot climates and high physical activities because they have high air permeability, high moisture transfer and dry quickly. Air permeability and thermal properties are improved by combining lyocell yarn with polyester technical yarns. Combining Lyocell or cotton with technical yarns deteriorates the moisture management properties and increases the drying times [24]. Sampath et al. investigated the thermal comfort properties of microdenier polyester filament, filament polyester and spun polyester, blends of polyester and cotton, and 100% cotton. The test results showed that the finishing treatment for moisture management resulted in higher thermal conductivity and thermal absorptivity, lower wet thermal resistance and improved water vapour permeability for all the fabrics studied. It was observed that microdenier polyester fabrics offered faster heat transfer, faster evaporation of sweat from the skin through the fabric and also a cooler feel at first touch [25]. Gericke and Pol investigated the selected comfort properties of cotton, regenerated bamboo and viscose rayon. No empirical evidence was found that regenerated bamboo fibres are superior to cotton and viscose fibres in terms of thermal and moisture management properties [26]. Gun investigated the thermal comfort properties of plain knitted fabrics made from modal viscose yarns with microfibers and conventional fibres. It was found that fabrics made of microfibers have lower air permeability, higher values of thermal conductivity, thermal absorptivity and maximum heat flux, and lower values of thermal resistance and thermal diffusivity compared to conventional fibres. Fabrics made of microfibers also provide a cooler feel compared to fabrics made of conventional fibres [27]. Lizák et al. focused on the thermal transport properties of polypropylene knitted fabrics. It was found that the rib structure is the most suitable for heat insulation due to the number of pores between the fibres and the low areal density of the fabric. It was demonstrated that heat loss is higher in a cardigan (tuck) structure [28]. In one of their studies on the thermal properties of fabrics made of natural and regenerated bamboo cellulose fibres, Majumdar et al. found that air and water vapour permeability increased but thermal conductivity decreased as the percentage of bamboo fibres increased [29]. Another study by Biviainyte et al. on double layer fabrics knitted from cotton, bamboo and four types of synthetic yarns found that fabrics knitted from a combination of cotton and synthetic yarns had a higher thermal conductivity coefficient than those knitted from a combination of bamboo and synthetic yarns. The knitted structure has the greatest influence on the thermal properties. Combined structures have a higher thermal conductivity coefficient than plain plated structures [30]. Jhanji et al. found that the thermal resistance and thermal conductivity of plated fabrics knitted with polyester yarn of different linear density in the inner layer decreased as the polyester yarn became finer. Similar trends were observed for fabrics knitted with viscose yarn of different linear density in the inner layer and cotton yarn of fixed linear density in the outer layer. It was also found that as the loop length of the fabric increases, the thermal resistance and thermal conductivity decrease, regardless of the fiber type of the inner layer, i.e., polyester, viscose or nylon [31]. Kaplan and Yilmaz investigated the thermal conductivity and air permeability of double-sided knitted fabrics combining functional yarns such as Thermosoft^®^, Nilit Heat^®^, Vi-loft^®^ and wool with standard polyester (PET) and polypropylene (PP) in a false rib structure. The results show that filament yarns have lower areal density and thickness, while staple yarns have higher air permeability. Despite the general trend of using hydrophobic fibers for the inner layers of knitted fabrics, wool and other functional man-made fibers such as Thermosoft^®^, Heat^®^, and Viloft^®^ can also be used for the inner surfaces that come into contact with the skin to improve sensory performance [32]. In their study, Kumar et al. investigated nine different types of knitted fabrics made from machine-spun yarns of Eri-silk, worsted wool, and bamboo fibres in three different knitted structures such as single jersey, single pique, and honeycomb. The thermo-physiological properties such as thermal conductivity, thermal resistance, thermal absorptivity, air permeability and water vapor permeability were investigated. It was found that the Eri-silk based knitted honeycomb structure was rated as better than the other samples [33]. 

The literature review shows that mainly knitted fabrics made of cotton [15,21,24,25,26,29,30,31], polyester [15,19,24,25,30,31,32] and regenerated cellulose [19,22,24,26,27,29,30,31] have been studied. A few studies were conducted with polyacrylonitrile [16,17,18], wool [16,32] and polypropylene [26,32], while individual studies were conducted with silk [33]. In terms of yarn parameters, the yarn linear density was mostly varied. In some cases, the influence of yarn twist [16,21] and plying of different yarns [22] were also studied. Single, rib and interlock knits produced on circular knitting machines have been studied most frequently [15,16,17,18,19,20,21,22,23,24,25,26,27,28,29,31]. In some cases, different loop lengths were designed for each knitted structure [17,18,22,27]. Thermal properties were most commonly tested using Alambeta and Permatest devices. Individual tests have been carried out based on specially designed devices [17,30].

## 3. Experimental

In order to design a multipurpose knitted structure suitable for indoor and outdoor work and leisure as well as sports activities, the aim of the research was to investigate the thermal and air permeability properties of different single and double knitted structures made from different yarns on a 12-gauge knitting machine to achieve the optimal material and structural functionalization of knitted fabrics. For the present study, yarns/materials and plain, miss and tuck knitted structures were selected, which, according to the literature reviewed, have not been studied in detail for thermophysiological comfort by other researchers according to the literature reviewed. In addition, flat knitting technology was chosen for the fabrication of the samples, which is suitable for both indoor and outdoor athleisure fabrication and is more flexible in terms of patterning and adjustment of structural parameters of knitted fabrics compared to circular knitting technology, which is mostly used for finer body-hugging athleisure production.

### 3.1. Sample Preparation

#### 3.1.1. Yarn Selection

Commercially available materials most commonly used in the manufacture of knitted sportswear and casual outerwear were selected for the study: cotton, wool, PAN and a wool/PAN blend. In addition, PA filament was used as a possible material for a more porous knit for active sportswear. 

Yarns were designated as follows: Y1–100% cotton; Y2–100% wool; Y3–100% PAN; Y4–50% Wool/50% PAN; Y5–100% PA.

#### 3.1.2. Knitted Samples Preparation

The knitted fabrics were produced on a flat knitting machine, a Shima Seiki SES 122 RT, gauge 12E in five structures: plain single jersey, single miss, single tuck/single piqué, plain double jersey (all needles in function) and Milano rib under the same cam settings. 

Knitted structures were designated as follows: SP-single plain; SM-single miss; ST-single tuck; DP-double plain; DM-double Milano. The combinations of yarn and structure designations were used for the knitting samples, i.e., cotton single jersey-Y1 SP. Samples of 100% cotton (Y1) and 100% PA filament (Y5) were knitted with double threads while samples of 100% wool (Y2), 100% PAN (Y3) and wool/PAN blend yarn (Y4) were knitted with single threads (Table 1 and Table 2).

After knitting, the samples were relaxed for 48 h under standard conditions before testing. The yarn paths and photographs of the tested knitted structures are shown in Table 1. 

### 3.2. Testing Methods

#### 3.2.1. Yarn Testing

Within the scope of the experiments presented in this manuscript, a number of yarn properties were tested: yarn linear density, twist, evenness, hairiness and tensile properties. 

The yarn linear density was determined using the skein method, following the procedure described in ISO 2060 [34]. The number of twists was determined on a torsion meter-Twist tester (Mesdan lab). The determination was conducted using the untwist/retwist method [35]. The coefficient of mass variation, a parameter that characterizes the yarn’s unevenness, was measured using the Keisokki evenness tester 80, type B. During the measurement, the sensibility level was set to +50/+50/+200 (thin places/thick places/neps). The number of protruding hairs was measured using the Zweigl G 565 hairiness tester. For the measurement, the following lengths of protruding hairs were defined: 2, 4, 6 and 8 mm. The testing speed was set to 50 m/min. Finally, the yarn tensile properties (including breaking elongation and tenacity) were measured on the dynamometer Statimat M (Textechno). The measurements were performed following ISO 2062 and using method B: automatic. The yarn samples were taken directly from the conditioned packages [36].

The characteristics of the yarns are listed in Table 2: material composition, linear density, and twist, tenacity and elongation at break as well as the number of threads used to knit the samples on the 12-gauge knitting machine to achieve appropriate fabric density and feel.

#### 3.2.2. Knitted Fabric Testing

The basic structural parameters of the knitted fabric affecting the thermal properties were determined as fabric thickness, horizontal density and vertical density, and mass per unit area. The fabric thickness was measured according to the standard ISO 5084 under 20 g/cm^2^ pressure with 0.01 mm accuracy [37]. The horizontal and vertical densities (Dh and Dv) were determined by counting the loops over a length of 50 mm. The densities were then converted to the number of loops per cm. The mass per unit area was measured using the specimen sized at 100 × 100 mm and by weighting them on an analytic balance Kern ALJ 220-4.

A sweating guarded hotplate (MTNW, Seattle, DC, USA) was used to measure the fabric heat resistance and water vapour resistance of textiles. The device itself consists of a metal plate attached to a conductive block, a heating device, sensors, a temperature controller, and the appropriate software. Its structure allows the simulation of the processes occurring next to the human skin and is related to the transfer of heat and sweat [38,39,40]. For the measurement of sweat transfer, the semipermeable membrane is placed on the heated plate and the sample is placed on the membrane. In this way, the transfer of sweat from human skin through the placed material is simulated. In this experiment, the tests were performed in accordance with ISO 11092 [41]. For the measurements of material heat resistance, the air temperature was set at 20 °C, relative humidity at 65%, and air velocity at 1 ms^−1^. For the measurement of water vapour resistance, the air temperature was set to 35 °C and the humidity to 40% of the relative humidity. In this case, the air velocity was also 1 ms^−1^.

The air permeability of the samples was measured using the air permeability tester Air-Tronic B. The measurements were performed according to EN ISO SIST EN ISO 9237 [42] with a constant pressure drop of 50 Pa. For the samples knitted from yarns Y1 (100% cotton), Y2 (100% wool), Y3 (100% PAN) and Y4 (wool-PAN blend), the test area was 20 cm^2^. For the sample knitted from Y5 (100% PA filament), the test area was 5 cm^2^ due to its porosity.

## 4. Results

### 4.1. Results of Yarn Testing

The results of yarn linear density and yarn twist measurements are shown in Table 2. It can be seen that the PA filament yarn (Y5) differs significantly from the other (worsted) yarns, not only by its smooth appearance but also by a much lower linear density, resulting in much more porous knitted structures.

The results of the measurements of the yarn tensile properties, consisting of elongation at break and tenacity, are shown in Table 2. Among the worsted yarns, the wool/PAN blend yarn shows the higher elongation at break followed by PAN and cotton yarn. The wool yarn has the least elongation. The filament yarn PA is clearly different from all the worsted yarns as it shows extreme elongation at break of more than 300%. PA filament shows the highest tenacity, followed by a much lower tenacity of 100% cotton and wool/PAN blend yarn. 100% wool and 100% PAN yarn show the lowest tenacity.

The results of the measurement of yarn evenness and hairiness are shown in Table 3. Only the measurements for worsted yarns were carried out (samples Y1–Y4). The results show that the yarn made from wool/PAN blend has the highest hairiness, followed by yarn made from 100% wool and 100% PAN. The cotton yarn is significantly smoother. The yarn made of 100% wool is the most uneven, followed by the yarns made of PAN and cotton. The yarn from the wool/PAN blend has the highest evenness of all yarns.

The cotton yarn has a much higher twist (661 m^−1^) than other worsted yarns (10–114 m^−1^), which leads to a strong skewing of the single structures and could thus affect the usability of the cotton yarn for the production of knitwear.

### 4.2. Results of Knitted Fabric Testing: Structural Parameters

Table 4 shows the results of the measurements of the structural parameters of knitted samples, which are horizontal density, vertical density, mass per unit area and fabric thickness. As expected, the values of vertical density are higher than the values of horizontal density for all samples. Single tuck structure, i.e., the single piqué from each analysed yarn, has the lowest horizontal and vertical density of all the structures tested, which means that the single piqué structure is the most porous of all the structures, due to the tuck loop in every other wale and course.

As expected, the mass per unit area ranges from 92.68 for a single plain structure of PA yarn to 471.54 for double plain wool/PAN structure. Single structures have lower mass than double structures for all types of knitted fabrics, which is in line with expectations. The greatest thickness was measured for the plain double structure in all the materials analysed, followed by the Milano rib structure. Among the single structures, the thickest is the single tuck structure, i.e., single piqué, while the thinnest is the plain single structure for all materials (Table 4).

Fabric thickness has a great influence on thermal resistance: the thicker the fabric, the more thermally resistant. At this point it should be noted that the thickness of the knitted fabric was measured according to the standard ISO 5084 under a pressure of 20 g/cm^2^, which means that the actual thickness of the unloaded knitted fabric is likely to be higher due to the voluminosity and compressibility of the examined knitted fabrics. On the other hand, the general trend in clothing is towards lighter end products [43]. When designing knitted sportswear or leisurewear, therefore, the choice of a single or double structure must be adapted to the intended use of the garment and the season or environmental conditions.

### 4.3. Results of Knitted Fabric Testing: Thermal Properties, Air Permeability, and Water Vapour Resistance

**Figure 1:**
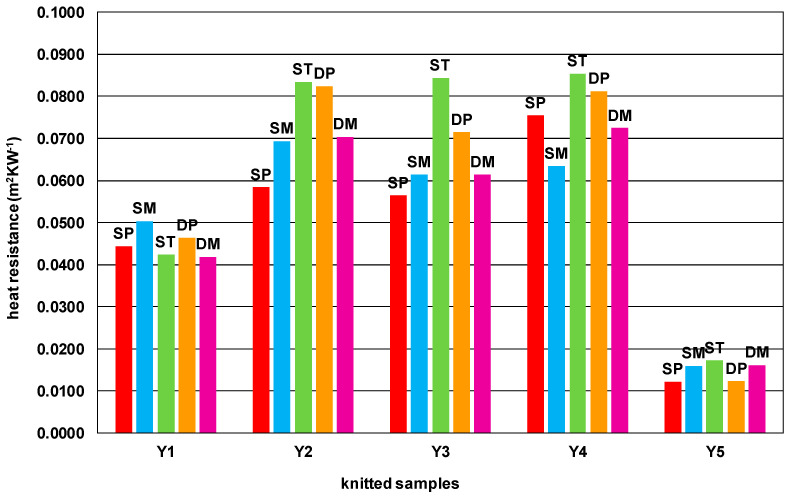
Heat resistance of knitted samples in different single and double structures.

Table 5 and Figure 1 show the results of the heat resistance measurements of the knitted samples measured on the hot plate.

All knitted samples made of PA filament yarn show the lowest heat resistance, while knitted samples made of 100% wool, 100% PAN and wool/PAN blend show the highest heat resistance. There are also greater differences in heat resistance between different structures knitted from these yarns (Figure 1). The heat resistance values of the structures knitted from 100% cotton and 100% PA filament yarns, which at the same time have lower linear densities and were knitted with double threads, show smaller differences. For wool, PAN and wool/PAN blend yarns, the single tuck/single piqué, and double plain structures are the most heat resistant.

**Figure 2:**
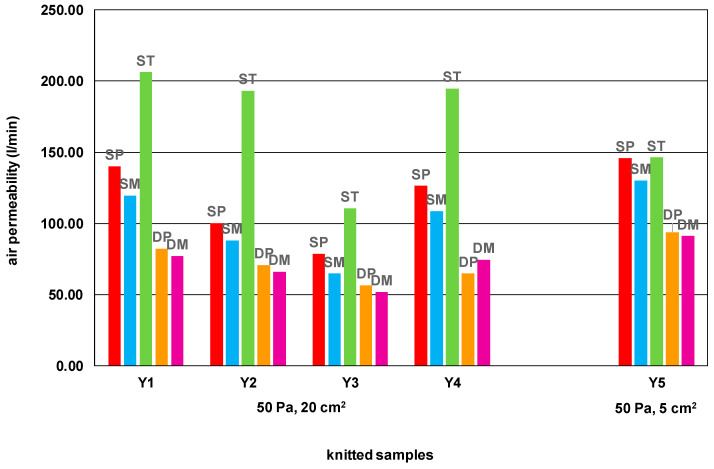
Air permeability of knitted samples in different single and double structures.

Table 6 and Figure 2 show the results of the air permeability measurements of the knitted samples.

As can be seen in Figure 2, single-knit structures show much greater air permeability compared to double-knit structures, which is due to their smaller thickness. The tuck structure stands out as having the lowest horizontal and vertical density among the single structures, despite having the greatest thickness, and the greatest porosity due to the enclosed tuck loops.

The air permeability results of knitted fabrics made of worsted yarns are not directly comparable with the air permeability results of filament yarns (Figure 2) because of the different measurement conditions. However, all yarns show a similar relationship between the air permeability of different structures, with the exception that the single tuck made of PA yarn is less noticeable compared to other structures. Presumably, the reason for this is the high porosity of all structures knitted from PA filament.

**Figure 3:**
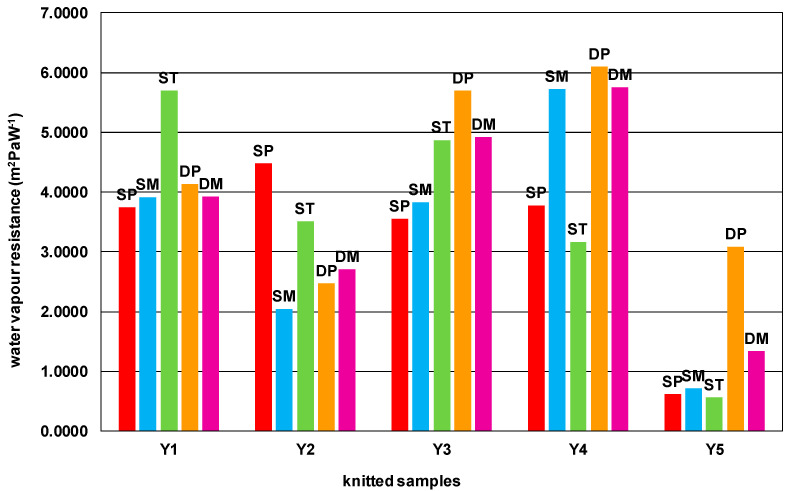
Water vapour resistance of knitted samples in different single and double structures.

Table 7 and Figure 3 show the results of the water vapour resistance measurements of the knitted samples. The measured values differ significantly and range from 0.5570 to 5.7542 m^2^ Pa W^−1^. As can be seen in Figure 3, knitted samples made of PA filament yarn show the lowest water vapour resistance. There are also differences in water vapour resistance between different structures. As expected, double structures show higher water vapour resistance than single structures, since their compact structure blocks a certain amount of water vapour that is supposed to pass through the material. Considering the obtained results, the single structures of 100% PA (especially when forming single plain, miss or tuck structures) are considered desirable for wearing during intense activities where the human body produces significantly more sweat. Such structures facilitate the perception of optimal thermophysiological comfort, as the sweat produced can be more easily removed from the body. This result is important for the behaviour of structures and is to be considered when developing material for a particular application.

## 5. Discussion

For the study, three single and two double basic structures were made from different yarns used commercially for sports and casual wear. The selected structures are suitable for indoor and outdoor activities, active sports and après-sports and leisure. The single plain structure is the simplest and most economical weft structure to produce and has maximum covering power. It usually has a recovery of 40% in width after stretching [44]. Due to its low-cost production, single jersey and its variants account for a large proportion of industrial production. All of the single plain knitted fabrics (made from all the selected yarns) had the least thickness. Compared to the other structures, their heat resistance was lowest when knitted from wool, PAN and PA yarns, and moderate when knitted from cotton and wool/PAN blend. All single plain knits have high air permeability due to their low thickness and rather open structure compared to single miss and double structures, which tend to be compact due to the underlying floats in single structure and folded wales in double structures. Air permeability is greater for single structures made of cotton and wool/PAN blend than for 100% wool and 100% PAN.

Missed structures show texture. The held loops give the impression of holes, and the floats produce a range of texture effects depending on their arrangement [45]. Structures containing floats tend to show dull horizontal lines. Knitted fabrics in miss-structures are narrower than equivalent all-knit fabrics because the wales are pulled closer together by the floats, which reduces the elasticity in width and improves the stability of the knitted fabric [44]. The miss-structure used in this study, also called weft locknit [44,46], only has short floats on the reverse side, but as can be seen in Table 1, it gives the impression of an inlaid or woven structure. All the single miss knits (from all the selected yarns) had the second lowest thickness. Compared to the other structures, their heat resistance was moderate when knitted from wool, PAN, wool/PAN blend and PA yarns and greatest when knitted from cotton yarn. All plain miss knits have lower air permeability compared to plain and tuck single structures and greater air permeability than double structures. This may be due to their relatively close structure compared to the other two single structures studied and their small thickness compared to double structures.

Tuck structures increase fabric width because tuck loops pull the held loops downward, spreading them outward and providing the additional yarn for width stretch. Fabric distortion and three-dimensional relief are caused by the accumulation of tuck loops, the displacement of wales and by the different number of tuck and knit loops per wale [44]. The most commonly encountered textured tuck structures have a cellular appearance on the reverse side of the fabric and a small piqué effect on the front. Tuck structures are usually flexible and have a bubbly surface [45]. The tuck structure used in this study was single piqué, also called Lacoste or Fred Perry [44]. Pique knit fabric has raised yarns that form various diamond-like shapes. This typically gives the fabric more body, making it perfect for polo-shirts, shirt dresses and more structured knit fashion apparel [47]. The examined single tuck structure had the greatest thickness of all the single structures due to the convexity of the loop arcs. It also had the greatest thermal resistance of all structures except those made of cotton. This could be due to the air trapped between the loops in the knitted structure. The tuck structure studied also showed the highest air permeability due to the porosity associated with the tuck loops and the low fabric density.

The double plain structure, or as it is also called, 1 × 1 rib, has a vertical cord appearance because the face loop wales tend to move over and in front of the reverse loop wales. Relaxed 1 × 1 rib is theoretically twice as thick and half as wide as an equivalent plain fabric, but has twice as much recoverable stretch in width and a heavier structure. The structure is elastic, form-fitting, and retains heat better than plain structures [44]. In this study, the double plain structure or 1 × 1 rib had the greatest thickness of all the structures due to the overlapping of the front and reverse loop wales. The heat resistance is highest in the knits of wool, PA and wool/PAN blend yarn. It is moderate in the knits made of cotton yarn and lowest in the knits made of PA filament. The double plain structure is less permeable to air compared to the three single structures analysed, which is due to its thickness.

Milano rib is a non-jacquard double structure [44]. The fabric shows courses of small loops on both sides of the fabric, alternating with courses of slightly larger loops, with the upper arcs of adjacent loops perfectly aligned as in 1 × 1 rib fabric. The structure shows a higher horizontal density and a lower vertical density than the other double structures studied, e.g., double plain structure or 1 × 1 rib. The fabric also has a lower thickness than the double plain structure. The heat resistance is lower than with a double plain structure for all the materials studied. Milano rib structures made of wool, PAN and wool/PAN blend have the highest heat resistance, while the one made of PA has the lowest. At the same time, Milano rib structures have the lowest air permeability of all the structures studied, except for the one made of wool/PAN blend, which is slightly less permeable than the double plain structure.

As we can conclude from the discussion above, the structure of the knitted fabrics (single plain, single miss, single tuck, double plain, and double Milano rib) certainly has an impact on comfort, heat resistance and air permeability. However, the structure itself is impossible to quantify and process statistically.

Certain elements of knitted fabric (horizontal density, vertical density, mass per unit area and thickness) can be quantified (Table 4). When the results of horizontal density and vertical density are statistically analysed, the correlation of the variables of horizontal density and vertical density with heat resistance is not statistically significant. Similar results are obtained from the correlation analysis of horizontal density, vertical density and air permeability. 

However, the effect of thickness on heat resistance is moderate to strongly correlated and proportional. As the thickness of the knitted fabric increases, the heat resistance increases. Mass per unit area is strongly correlated with heat resistance, more so than thickness (Table 8). It is enough to look at Figure 1 to see that the properties of the yarns have a noticeable effect on heat resistance. The correlation analysis confirms that the correlation coefficient between the linear density of the yarns (Table 2) and the heat resistance of the different knitted fabrics (Table 5) is high for each knitted structure (Table 8).

In studies, the little-observed factor of the hairiness of the yarn (Table 3) shows a significant impact on heat resistance. Interestingly, in addition to the number of protruding fibers, the length of the protruding fibers also has a statistically decisive influence (Table 8). The number of the shortest protruding fibers shows the strongest correlation with heat resistance. To increase the heat resistance, it is important to increase the number of protruding fibers.

The influence of thickness on the air permeability of knitted fabrics is medium to high and inversely proportional. As the thickness of the knitted fabric increases, the air permeability decreases, especially in double structures. The exception is a single tuck fabric, where the greatest porosity is influenced more by the shape of tuck loops than the thickness (Table 9).

It is similar when a correlation of air permeability with mass per unit area is observed. Mass per unit area also has a medium effect on air permeability, which is higher for double-knitted structures (Table 9).

The correlation coefficient between the linear density of the yarn (Table 2) and the air permeability of the different knitted fabrics is high and inversely proportional for each structure, except for the single tuck, where the structure and the shape of the loop are again more important (Table 9).

The hairiness of the yarn (Table 3) also shows a significant influence on air permeability. In addition to the number of protruding fibers, the length of the protruding fibers also has a statistically decisive influence (Table 9), although the influence is inversely proportional. To increase air permeability, it is important to reduce the number of protruding fibers. The hairiness of the yarn is again least important for single tuck fabric where the shape of the tuck loop has the greater influence.

The correlation coefficient between the linear density of the yarn (Table 2) and the water vapour resistance is relatively weak to moderate. Similar correlation coefficients were obtained for yarn hairiness. A positive correlation is observed for both properties of the yarn, i.e., the finer the yarn, the lower will be the water vapor resistance. Lower yarn hairiness, especially of the shortest and most numerous protruding fibers (2 mm) will result in lower water vapour resistance. Moderate to high positive correlation coefficients between the thickness and mass per unit area and water vapour resistance were obtained for all knitted structures. Higher thickness or mass per unit area will result in higher water vapour resistance. Again, the shape of the tuck loop is more important than the yarn parameters or the thickness of knitted material (Table 10).

At the same time, air permeability and heat resistance are inversely proportional and moderately strongly correlated, except for the single tuck fabric, where the structure and shape of the tuck loop is more important (Table 11).

From the correlation analysis, it can be concluded that yarn linear density, yarn short fibers hairiness, and mass per unit area of knitted fabric have the greatest impact on heat resistance (Table 8). The relationship between heat resistance and yarn hairiness and mass per unit area can be expressed by the multiple linear regression equation (Table 12). The share of variance of the dependent variable (heat resistance) interpreted by the model is very high (R^2^) for all knitted structures. The model is therefore representative. The statistical significance of the model is confirmed by *p*-value, significance F < 0.05 for all regression models.

The yarn linear density, the yarn hairiness of the longer protruding fibers, and the thickness of the knitted fabric have the greatest impact on air permeability, except in the case of tuck structures, where the shape of the tuck loop allows higher air permeability and all other parameters become less significant (Table 9). However, a statistically significant model can be constructed only for the double plain structure: Ap = 95.55241537 − 0.280261395 X_1_ − 0.052690916 X_2_, R^2^ = 0.96335, significance F= 0.03664, where air permeability (Ap) is the dependent variable, X_1_ is the yarn linear density, and X_2_ is the yarn hairiness.

A different result was obtained for water vapour resistance, on which the mass and thickness of the knitted fabric have the strongest influence. A statistically significant model can be constructed only for the single plain structure: Ret = −0.534779 − 3.13649 X_1_ + 0.032617 X_2_, R^2^ = 0.96147, significance F= 0.03852, where water vapor resistance (Ret) is the dependent variable, X_1_ is the thickness, and X_2_ is the mass per unit area.

## 6. Conclusions

It can be stated that the single tuck structure, i.e., single piqué, made of wool, PAN or wool/ PAN blend, has the highest heat resistance, medium water vapour resistance, and at the same time the highest air permeability, which favours it for use for sportswear when body heat should be preserved, as well as après-sports and casual wear because of the comfort it could provide. Along with the highest hairiness of these yarns, a comfortable feel can be expected, but at the same time, pilling can occur. Single piqué made from 100% cotton yarn has a lower heat resistance, which qualifies it for summer clothing. The disadvantage of using this yarn could be the high twist and the resulting skewness of the structure.

The double plain knitted structure has the second highest heat resistance for all materials and the highest water vapour resistance. At the same time, its air permeability is low compared to other fabrics analysed, which, combined with the highest thickness of all the structures and materials studied, could make it the most suitable knitted fabric for outdoor use. The double plain structure is also very stretchy, which ensures a good fit to the body.

The single plain structure has the lowest heat resistance and the lowest water vapour resistance, which qualifies this structure for rapid loss of body heat, i.e., for clothing for active training. This structure has moderate air permeability, and the combination of a single plain and a single tuck structure would increase air permeability and give an ideal structure of knitted fabric for active training and rapid body heat loss.

Statistical analysis shows that when designing knitwear, it is important to consider not only the structure itself but also the raw material composition of the yarn, which is strongly correlated with thermal resistance and air permeability. A less considered factor, the hairiness of the yarn, correlates strongly with thermal resistance, water vapour resistance, and air permeability. To increase thermal resistance, it is important to increase the number of protruding fibers. Conversely, to increase air permeability, it is important to decrease the number of protruding fibers.

All the knitted structures studied were least air permeable when made from 100% PAN, which could affect their comfort. All structures made from PA filament showed significantly lower heat resistance, the lowest water vapour resistance, and high air permeability. The studied PA filament yarn is very stretchy and has high tenacity. All the studied structures are thinnest when made from the PA filament, which contributes to their lightness. It can be concluded that the PA filament is best suited for the use of sportswear in hot indoor or outdoor climates.

## Figures and Tables

**Table 1:** Single and double knitted structures selected for the knitted samples.

	Single Structures			DOUBLE Structures	
**Appearance** **front**	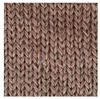	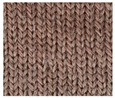	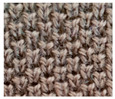	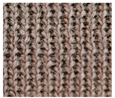	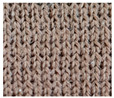
**Appearance** **back**	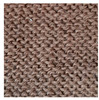	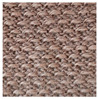	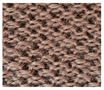	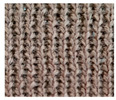	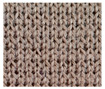
**Yarn path**	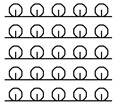	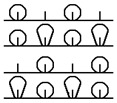	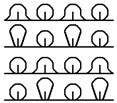	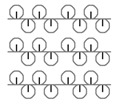	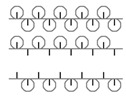
**Structure**	Single plain	Single miss	Single tuck	Double plain	Double Milano
**Designation**	SP	SM	ST	DP	DM

**Table 2:** Yarn designation and characteristics.

Yarn Designation	Yarn Material Composition	Yarn Linear Density (tex)	Yarn Twist (m^−1^)	Number of Threads for Knitting	Yarn Tenacity (cNtex^−1^)	Yarn Elongation at Break (%)
**Y1**	100% Cotton	29.1	661	2	13.90	5.20
**Y2**	100% Wool	68.6	88	1	5.25	2.90
**Y3**	100% PAN (bouclé)	72.3	10	1	4.66	12.55
**Y4**	50% Wool 50% PAN	71.4	114	1	12.23	14.30
**Y5**	100% PA (filament)	15.4	-	2	40.76	301.27

**Table 3:** Yarn evenness and hairiness.

Yarn Designation	Yarn Material Composition	Yarn Evenness–CV_mass_ (%)	Yarn Hairiness			
			n1 (2 mm)	n2 (4 mm)	n3 (6 mm)	n4 (8 mm)
**Y1**	100% Cotton	11.75	952	147	23	5
**Y2**	100% Wool	19.04	1604	494	156	88
**Y3**	100% PAN (bouclé)	12.04	1350	718	346	283
**Y4**	50% Wool 50% PAN	9.47	1804	535	174	95
**Y5**	100% PA (filament)	-	-	-	-	-

**Table 4:** Knitted samples designation and structural properties.

Material Composition	Fabric Designation	Knitted Structure	Horizontal Density (cm^−1^)	Vertical Density (cm^−1^)	Mass per Unit Area (gm^−2^)	Thickness (mm)
**Y1** **100% Cotton**	**Y1 SP**	single plain	7.98	8.32	260.38	1.23
	**Y1 SM**	single miss	7.88	8.32	287.17	1.37
	**Y1 ST**	single tuck/single piqué	5.32	6.68	267,35	1.54
	**Y1 DP**	double plain	6.40	9.08	433.40	1.92
	**Y1 DM**	double Milano rib	7.26	8.82	430.49	1.80
**Y2** **100% Wool**	**Y2 SP**	single plain	6.00	8.00	238.19	0.98
	**Y2 SM**	single miss	6.70	7.76	281.49	1.22
	**Y2 ST**	single tuck/single piqué	4.66	6.64	286.40	1.85
	**Y2 DP**	double plain	5.76	8.76	438.49	2.00
	**Y2 DM**	double Milano rib	6.54	8.42	453.85	1.77
**Y3** **100% PAN**	**Y3 SP**	single plain	5.80	7.90	233.73	1.22
	**Y3 SM**	single miss	6.36	7.92	267.36	1.52
	**Y3 ST**	single tuck/single piqué	4.62	5.72	250.79	1.78
	**Y3 DP**	double plain	5.16	8.72	459.36	2.28
	**Y3 DM**	double Milano rib	6.08	8.64	453.15	2.01
**Y4** **Wool/PAN**	**Y4 SP**	single plain	5.88	7.64	234.20	1.00
	**Y4 SM**	single miss	6.44	7.62	263.41	1.24
	**Y4 ST**	single tuck/single piqué	4.68	6.80	299.42	1.89
	**Y4 DP**	double plain	5.82	8.88	471.54	2.06
	**Y4 DM**	double Milano rib	6.44	8.08	455.06	1.76
**Y5** **100% PA**	**Y5 SP**	single plain	5.52	8.20	92.68	0.58
	**Y5 SM**	single miss	5.88	6.36	132.51	0.77
	**Y5 ST**	single tuck/single piqué	3.88	3.86	132.51	1.40
	**Y5 DP**	double plain	5.36	5.88	231.54	1.70
	**Y5 DM**	double Milano rib	6.02	5.16	216.42	1.44

**Table 5:** Heat resistance of knitted samples.

Yarn/Structure		Heat Resistance (m^2^ KW^−1^)				
		SP	SM	ST	DP	DM
		Single Plain	Single Miss	Single Tuck	Double Plain	Double Milano rib
**Y1**	**100% Cotton**	0.0444	0.0504	0.0424	0.0464	0.0419
**Y2**	**100% Wool**	0.0584	0.0694	0.0834	0.0824	0.0704
**Y3**	**100% PAN**	0.0564	0.0614	0.0844	0.0714	0.0614
**Y4**	**Wool/PAN**	0.0754	0.0634	0.0854	0.0812	0.0724
**Y5**	**100% PA**	0.0122	0.0160	0.0172	0.0123	0.0162

**Table 6:** Air permeability of knitted samples.

Yarn/Structure		Air Permeability (l min^−1^)				
		SP	SM	ST	DP	DM
		Single Plain	Single Miss	Single Tuck	Double Plain	Double Milano rib
**Y1**	**100% Cotton**	140.12	119.72	206.14	82.44	77.14
**Y2**	**100% Wool**	100.40	88.20	193.30	70.92	66.20
**Y3**	**100% PAN**	78.76	64.68	110.66	56.78	51.84
**Y4**	**Wool/PAN**	126.18	108.74	194.86	64.90	74.60
**Y5**	**100% PA**	145.88	130.18	146.28	93.92	91.02

**Table 7:** Water vapour resistance of knitted samples.

Yarn/Structure		Water Vapour Resistance (m^2^ Pa W^−1^)				
		SP	SM	ST	DP	DM
		Single Plain	Single Miss	Single Tuck	Double Plain	Double Milano rib
**Y1**	**100% Cotton**	3.7448	3.9135	5.6966	4.1322	3.9222
**Y2**	**100% Wool**	4.4758	2.0459	3.5071	2.4678	2.7070
**Y3**	**100% PAN**	3.5522	3.8274	4.8722	5.6936	4.9208
**Y4**	**Wool/PAN**	3.7760	5.7246	3.1658	6.0944	5.7542
**Y5**	**100% PA**	0.6112	0.7075	0.5570	3.0855	1.3329

**Table 8:** The correlation coefficients of heat resistance and selected yarn and fabric properties.

Knitted Sample Structure	Correlation Coefficients						
	Linear Density	Yarn Hairiness (Length of Protruding Fibres)				Thickness	Mass per Unit Area
		2 mm	4 mm	6 mm	8 mm		
**Single plain**	0.89241	0.98688	0.81619	0.63217	0.48558	0.63803	0.81236
**Single miss**	0.89081	0.96882	0.83147	0.66089	0.53297	0.79345	0.92944
**Single tuck**	0.99334	0.95358	0.95406	0.82584	0.70488	0.98268	0.82534
**Double plain**	0.95409	0.98832	0.88101	0.70842	0.56797	0.78978	0.90537
**Double Milano rib**	0.95421	0.99198	0.87418	0.69593	0.55038	0.70619	0.90318

**Table 9:** The correlation coefficients between air permeability and selected yarn and fabric properties.

Knitted Sample Structure	Correlation Coefficients						
	Linear Density	Yarn Hairiness (Length of Protruding Fibres)				Thickness	Mass per Unit Area
		2 mm	4 mm	6 mm	8 mm		
**Single plain**	−0.80487	−0.58298	−0.89139	−0.93040	−0.91987	−0.50103	−0.44484
**Single miss**	−0.81007	−0.60602	−0.90414	−0.94283	−0.93351	−0.71800	−0.50944
**Single tuck**	−0.06720	0.30399	−0.27054	−0.52998	−0.66410	0.14010	0.50699
**Double plain**	−0.94761	−0.84736	−0.98875	−0.94508	−0.87497	−0.97376	−0.83646
**Double Milano rib**	−0.81155	−0.67561	−0.90661	−0.92479	−0.90892	−0.93086	−0.76062

**Table 10:** The correlation coefficients between water vapour resistance and selected yarn and fabric properties.

Knitted Sample Structure	Correlation Coefficients						
	Linear Density	Yarn Hairiness (Length of Protruding Fibres)				Thickness	Mass per Unit Area
		**2 mm**	**4 mm**	**6 mm**	**8 mm**		
**Single plain**	0.74784	0.90664	0.67931	0.49148	0.36833	0.79575	0.95664
**Single miss**	0.56481	0.72480	0.55035	0.43234	0.33876	0.67858	0.73407
**Single tuck**	0.37182	0.53672	0.43834	0.39050	0.37734	0.34807	0.70002
**Double plain**	0.47280	0.45394	0.55580	0.57826	0.55864	0.6553	0.54081
**Double Milano rib**	0.68288	0.761314	0.70517	0.62598	0.54825	0.7408	0.77297

**Table 11:** Correlation coefficient of air permeability and heat resistance for different structures.

Knitted Sample Structure	Correlation Coefficient
Single plain	−0.51528
Single miss	−0.69783
Single tuck	0.02073
Double plain	−0.87624
Double Milano rib	−0.71627

**Table 12:** Relationship between heat resistance (R), yarn hairiness (X_1_) and mass per unit area (X_2_) for different knitted structures.

Knitted Sample Structure	Multiple Linear Regression Equations	R^2^	Significance F
Single plain	R = 0.0120697 + 3.24855 × 10^−5^ X_1_ + 9.05167 × 10^−7^ X_2_	R^2^ = 0.97393	Signif. F = 0.02606
Single miss	R = 0.0018770 + 2.00342 × 10^−5^ X_1_ + 1.09454 × 10^−4^ X_2_	R^2^ = 0.96437	Signif. F = 0.03562
Single tuck	R = 0.0699356 + 7.40042 × 10^−5^ X_1_ − 3.70694 × 10^−4^ X_2_	R^2^ = 0.97055	Signif. F = 0.02944
Double plain	R = 0.0201838 + 4.54164 × 10^−5^ X_1_ − 3.27122 × 10^−5^ X_2_	R^2^ = 0.97837	Signif. F = 0.02162
Double Milano rib	R = 0.0228920 + 3.71459 × 10^−5^ X_1_ − 3.19884 × 10^−5^ X_2_	R^2^ = 0.98672	Signif. F = 0.01327

## Data Availability

Data is contained within the article.
